# Enhancing Palladium Recovery Rates in Industrial Residual Solutions through Electrodialysis

**DOI:** 10.3390/membranes13110859

**Published:** 2023-10-26

**Authors:** Pauline Zimmermann, Önder Tekinalp, Øivind Wilhelmsen, Liyuan Deng, Odne Stokke Burheim

**Affiliations:** 1 Department of Energy and Process Engineering, Norwegian University of Science and Technology (NTNU), NO-7491 Trondheim, Norway; pauline.zimmermann@ntnu.no; 2 Department of Chemical Engineering, Norwegian University of Science and Technology (NTNU), NO-7491 Trondheim, Norway; onder.tekinalp@ntnu.no (Ö.T.); liyuan.deng@ntnu.no (L.D.); 3 Department of Chemistry, Norwegian University of Science and Technology (NTNU), NO-7491 Trondheim, Norway; oivind.wilhelmsen@ntnu.no

**Keywords:** electrodialysis, anion exchange membrane, palladium, recovery, energy consumption

## Abstract

Palladium is a vital commodity in the industry. To guarantee a stable supply in the future, it is imperative to adopt more effective recycling practices. In this proof-of-concept study, we explore the potential of electrodialysis to enhance the palladium concentration in a residual solution of palladium recycling, thus promoting higher recovery rates. Experiments were conducted using an industrial hydrochloric acid solution containing around 1000 mg/L of palladium, with a pH below 1. Two sets of membranes, Selemion AMVN/CMVN and Fujifilm Type 12 AEM/CEM, were tested at two current levels. The Fujifilm membranes, which are designed for low permeability of water, show promising results, recovering around 40% of palladium within a two-hour timeframe. The Selemion membranes were inefficient due to excessive water transport. All membranes accumulated palladium in their structures. Anion-exchange membranes showed higher palladium accumulation at lower currents, while cation-exchange membranes exhibited increased palladium accumulation at higher currents. Owing to the low concentration of palladium and the presence of abundant competing ions, the current efficiency remained below 2%. Our findings indicate a strong potential for augmenting the palladium stage in industrial draw solutions through electrodialysis, emphasizing the importance of membrane properties and process parameters to ensure a viable process. Beyond the prominent criteria of high permselectivity and low resistance, minimizing the permeability of water within IEMs remains a key challenge to mitigating the efficiency loss associated with uncontrolled mixing of the electrolyte solution.

## 1. Introduction

**Figure 1:**
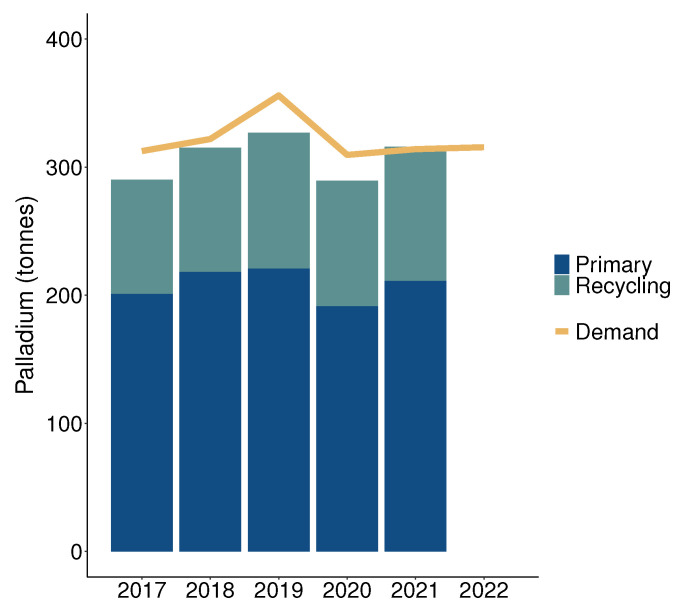
Global supply and demand of palladium from 2017 to 2022. Data are taken from [3]. For 2022, data were available for demand only.

Platinum group metals’ (PGMs) exceptional chemical properties, including catalytic activity, electric conductivity, and resistance to oxidation and corrosion, make them irreplaceable in various industries such as automobile, chemical engineering, petroleum, electrical, and electronics [1,2]. However, the occurrence of primary PGM ore is scarce within the Earth’s crust, with the predominant deposits primarily located in South Africa and Russia. Although researchers have endeavored to identify alternative materials that can partially replace PGMs in products like automobile catalysts, the overall consumption of PGMs remains high. More than 80% of the worldwide palladium demand is ascribed to the automotive sector [3], mostly to catalytic converters, which are critical in reducing harmful emissions from engines. The enforcement of stricter emission regulations, coupled with rapid growth of the new-energy automobile industry, is expected to further strain the supply of PGMs [4]. Figure 1 provides an overview of the global supply of palladium from primary and secondary sources compared to the demand in the years 2017 to 2022, revealing a deficit for the past five years. Given the high economic importance and elevated supply risk associated with PGMs, the European Commission has classified PGMs as critical raw materials [5]. To secure the palladium supply in the future, as well as strengthen strategic autonomy and responsible sourcing of raw materials, it is critical to seek the most effective recovery from secondary sources [6]. In 2021, secondary sources contributed to meeting one-third of the global palladium demand. Besides offering increased economic benefits, the recycling of PGMs enhances energy efficiency and environmental sustainability [7]. However, the methods used for separation often rely heavily on chemicals and fall short of achieving a 100% recovery rate due to significant dilution factors. Achieving effective and comprehensive separation of metals at low concentrations is a crucial objective for optimization in the fields of metal recovery and refining [8,9,10].

In hydrochloric acid media, palladium exists in the aqueous phase as strong chloride complexes [11]. Specifically, at HCl concentrations above 1 M, the predominant palladium species is ${\left[{\mathrm{PdCl}}_{4}\right]}^{2-}$ [12,13]. The equilibrium redox potential for palladium in chloride media can shift the oxidation state according to [14]:

(1) $${\left[{\mathrm{PdCl}}_{6}\right]}^{2-}+2e^{-}⇌{\left[{\mathrm{PdCl}}_{4}\right]}^{2-}+2{\mathrm{Cl}}^{-}\;-1.29V$$

The different oxidation states can be manipulated through selective oxidation or reduction, taking into account both kinetic and thermodynamic factors. This manipulation allows to achieve separation using various techniques. For instance, precipitation or ion-exchange can be employed to remove Pd(IV), and complexation can be used to remove Pd(II). Several reviews have been presented on separation and purification methods for PGMs from aqueous solutions, including solvent extraction, membranes separation, supercritical fluids, solid-phase extraction, photoreduction, and electrochemical methods [1,2,4,5,15]. In the context of hydrometallurgy, membrane separation techniques exhibit superior performance compared to treatment methods like precipitation, adsorption, coagulation-flocculation, and solvent extraction in several crucial aspects. Some benefits of membrane separation techniques include separation and concentration factors, mass transfer rate, single-step operation with reduced chemical consumption, and the absence of solid waste or sludge containing hazardous or unstable components that necessitate disposal [5,16,17]. For the selective recovery of valuable components, the remarkable selectivity provided by functionalized membranes is a key factor [18]. In general, electrodialysis can effectively handle significant volumes of effluents containing low concentrations of specific ions in a relatively short timeframe [19]. Typically, for cost-effective operation of electrodialysis, salt concentrations ranging between 0.01 and 0.5 M are considered feasible, although this may vary depending on the specific characteristics of the process and its components [20]. Liquid membranes, in particular, have been the focus of significant research activity for PGM recovery [1,21,22,23,24,25,26,27,28,29]. A liquid membrane can be defined as a layer of organic solvent that separates two aqueous solutions, containing carriers that facilitate the transport of ions from one solution to the other [30]. Remarkably high palladium recovery rates of up to 100% have been reported using liquid membranes [25]. However, certain limitations hinder the performance of liquid membrane extraction, including slow mass transport rates and the long-term stability of the membranes, which are susceptible to fouling, carrier molecule leakage, solvent phase loss, support swelling, and chemical degradation. To address these challenges, the application of liquid membranes in an electrodialysis setup has shown promise, as the direct application of an electric field facilitates the efficient removal of metals from the organic phase [28,30,31]. In a recent study, utilizing an electrodialysis setup, a peak palladium flux of 120 $\mu \mathrm{mol}\;\cdot \;m^{-2}s^{-1}$ was achieved through a liquid membrane, with an initial concentration of 0.13 M in the feed solution [31]. In electrodialysis, an electric potential is established across the cell, triggering electrochemical processes at the electrodes and creating an electric field. Consequently, cations move towards the cathode by traversing the cation-exchange membrane (CEM), while conversely, anions migrate towards the anode, transferring through the anion-exchange membrane (AEM). Thereby, ions accumulate in the concentrate compartment, while the diluate compartment is depleted of ions. The permselectivity determines the degree to which the IEMs selectively allow ions of opposite charge (counter-ions) to permeate while repelling ions of like charge (co-ions) [32]. Apart from their charge, the hydration energy of ions can be used to control their permeability. Ions have been demonstrated to transport a hydration shell that is both specific to the ion type and influenced by the properties of the membrane they traverse [33,34]. Consequently, enhancing membrane hydrophobicity is a strategy for tailoring membrane-ion selectivity, aiming to discriminate ions with larger hydration shells [35,36]. To the best of the authors’ knowledge, there are no studies available on the utilization of conventional electrodialysis for enhancing the palladium concentration in solutions. Therefore, this proof-of-concept study aims to explore the potential of electrodialysis for the recovery of palladium from a residual solution after a chemical palladium separation step. In this work, electrodialysis has been performed using an industrial solution containing hydrochloric acid and metal residuals, characterized by a pH below one and a palladium concentration of 1000 mg/L. According to Equation (1), palladium exists in this medium in its tetra- or hexachloric complex as a divalent anion. The high ratio of chloride and hydrogen ions to palladium ions compromises the current efficiency of the process significantly, as the available ions contribute to the charge transfer in the electrodialysis cell according to their concentration and mobility in the membranes [18,37]. Furthermore, the water transport through the membranes counteracts the increase in concentration. Consequently, the membrane’s capability to prevent the transport of water is of utmost importance. Accordingly, the objective of this study is to investigate the feasibility of increasing the palladium concentration in low-concentrated acidic residual solution using electrodialysis, and specifically to (1) compare the palladium recovery with two membrane pairs of varying water permeability, (2) determine the performance in terms of energy requirements, and (3) assess the impact of the current density on the process performance. The analysis will help to identify design strategies and membrane requirements for future research on separation tasks, including low concentrations of critical compounds in aqueous mixtures.

## 2. Materials and Methods

### 2.1. Experimental Setup

**Figure 2:**
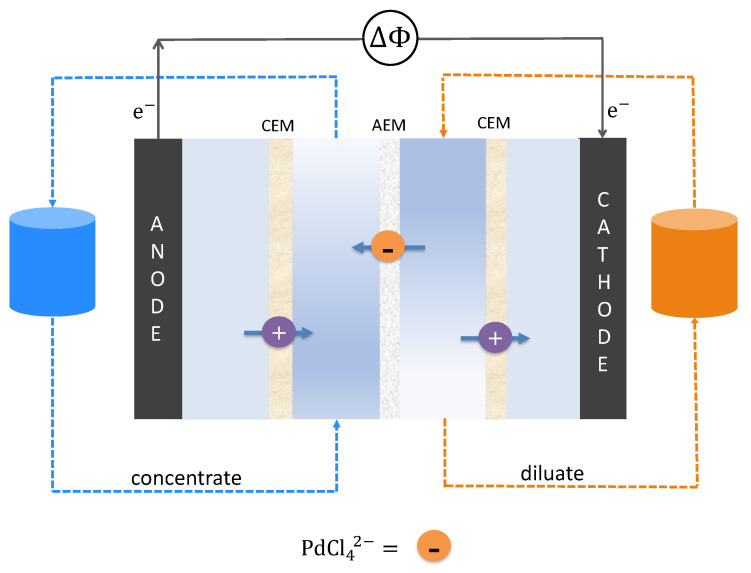
An illustration of the electrodialysis setup for palladium recovery. Pd is expected to predominantly exist as PdCl_4_^2−^ complexes, consequently facilitating its migration through the AEM.

The electrodialysis cell employed in this study is illustrated in Figure 2. A custom cross-flow electrodialysis stack was used, featuring a 9 × 4 ${\mathrm{cm}}^{2}$ electrode area. One AEM was sandwiched between two CEMs, dividing the cell into four distinct compartments. The electrodes were connected to a power supply. The compartments neighboring the electrodes contained rinse solution to facilitate the charge transfer. The process was run in batch mode, where one feed batch becomes gradually concentrated and the other depleted of ions.

### 2.2. Materials

An industrial barren solution was provided by K.A. Rasmussen with the properties listed in Table 1. The solution is the residual filtrate obtained subsequent to the separation and purification of palladium. The metal content was analysed using Inductively Coupled Plasma Optical Emission Spectroscopy (ICP-OES). The pH is quite low, owing to the leaching process involving noble metals in aqua regia, where a molar ratio of 1:3 nitric acid to hydrochloric acid is employed. Through the evaporation of nitric acid from the solution, a matrix predominantly composed of hydrochloric acid remains, which still contains a significant concentration of palladium as well as other dissolved precious metals at lower concentrations.

The desalination experiments were performed with two different commercially available AEM and CEM pairs, Selemion AMVN and CMVN (Eurodia Industries SAS, Pertuis, France) and Fujifilm Type 12 AEM and CEM (Fujifilm Manufacturing Europe B.V., Tilburg, The Netherlands). Some of the known membrane properties are listed in Table 2. The Fujifilm Type 12 membranes are recommended to be used within a pH range from 1 to 13 [38]. For Selemion membranes, a pH of 7 or below is recommended, as alkaline solutions may degrade the membranes [39]. The active membrane surface area was 36 ${\mathrm{cm}}^{2}$. Woven silicone/polyester spacers with integrated gaskets were supplied by FumaTech BWT GmbH (Bietigheim-Bissingen, Germany) with a thickness of 470 μm, a mesh size of 800 μm, and a shadow effect of 0.33, according to supplier information. Rinse solutions were prepared using distilled water and technical grade sodium sulfate (Na_2_SO_4_) provided by Honeywell International Inc. (Charlotte, NC, USA) Shenchen V6-6L peristaltic pumps (Baoding Shenchen Precision pump Co., Ltd., Baoding, China) recirculated the solutions. ICP-OES was used to analyze the palladium concentrations in the samples.

### 2.3. Methods

In the desalination experiments, two 150 mL batches of the industrial solution were recirculated at a flow rate of 100 mL/min. Spacers were introduced between the membranes to aid solution flow and mixing. End spacers were positioned between the electrodes and adjacent CEMs. Each batch was stirred with a magnetic stirrer. A 500 mL batch of 0.5 M sodium sulfate rinse solution was recirculated through the electrode compartments. Experiments were run at a constant current of either 0.5 A or 1 A for two hours. During the experiments, 25 mL samples were collected every 30 min and their palladium concentration was analyzed.

The palladium flux was calculated based on the concentration change in the concentrate compartment [41]:

(2) $$J_{Pd}=\frac{\Delta N_{Pd}}{A\Delta t}$$

where $\Delta N_{Pd}$ is the variation of the number of palladium ion equivalents in the concentrate compartment, *A* is the active area of the membrane, and Δt the time step. The water drag from the dilute to the concentrate compartment was neglected in the flux calculations.

The specific energy consumption, or work input, per equivalent of palladium recovered in the concentrate, $W_{Pd}^{spec}$, was calculated as follows:

(3) $$W_{Pd}^{spec}=\frac{UI\Delta t}{\Delta N_{Pd}}$$

where *U* is the electric potential and *I* is the electric current.

The current efficiency for palladium recovery, $R_{Pd}$, was defined by the ratio of palladium ion recovery in the concentrate to the number of coulombs transferred in the system [42]:

(4) $$R_{Pd}=\Delta N_{Pd}\frac{F}{Q}$$

where *F* is the Faraday constant, and Q=IΔt is the number of coulombs that has been transferred.

To assess the extent of palladium loss in the solutions attributed to scaling, the mass of palladium attached to the membranes was quantified after the experiments. The membranes were dissolved using aqua regia, with the application of heat to accelerate this process. Any residual matter that resisted dissolution was transferred to a crucible, where subsequent exposure to elevated temperature in an oven facilitated the removal of organic constituents. The resulting ashes were similarly subjected to dissolution using aqua regia. The palladium content in the resulting solutions was analyzed with ICP-OES. For elucidating the palladium species that can occur in the feed solution, a speciation model was run in *Visual MINTEQ* software (version 3.1, created by Jon Petter Gustafsson at KTH, Stockholm, Sweden) [43]. The Davies model [44] was employed to calculate activities in the electrolyte.

## 3. Results and Discussion

**Figure 3:**
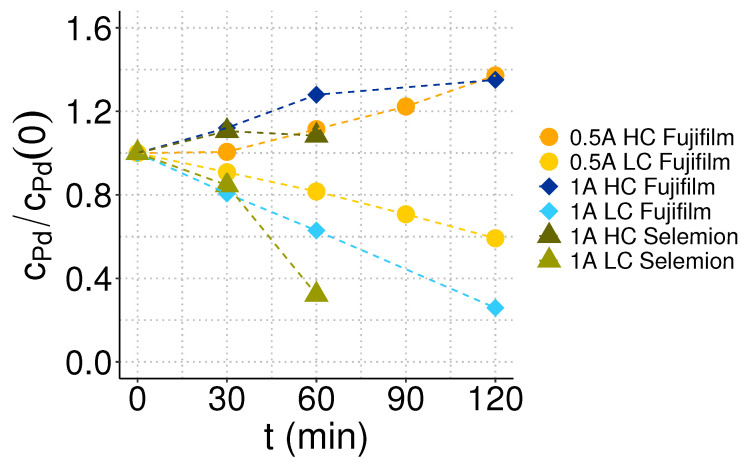
Concentration profiles of palladium in the concentrate (HC) and dilute (LC) compartments during electrodialysis at 1 A or 0.5 A. The experiment with Selemion membranes was aborted after 1 h due to excessive water transfer from the diluate to the concentrate compartment.

Figure 3 shows the degree of palladium removal and recovery from the diluate and concentrate batches during the electrodialysis treatment of palladium residual filtrate at different process conditions. In the initial approach, the desalination performance with two membrane pairs, Selemion CMVN/AMVN and Fujifilm Type 12 CEM/AEM, was compared by drawing a current of 1 A, which corresponds to a current density of about 28 mA/${\mathrm{cm}}^{2}$. Current-voltage curves were recorded for both membrane pairs within the electrodialysis cell to assess the operational current regime. The experimental procedure and obtained plots are provided in Appendix A. The current-voltage curve for the Fujifilm membranes shows a linear behavior, suggesting that the limiting current density is not reached within the tested electric current range between 0 an 1.1 A. For the Selemion membranes, the slope of the current-voltage curve decreases around a current value of 0.5 A. The decreasing potential with increasing current suggests that the overlimiting current regime has been reached.

**Figure 4:**
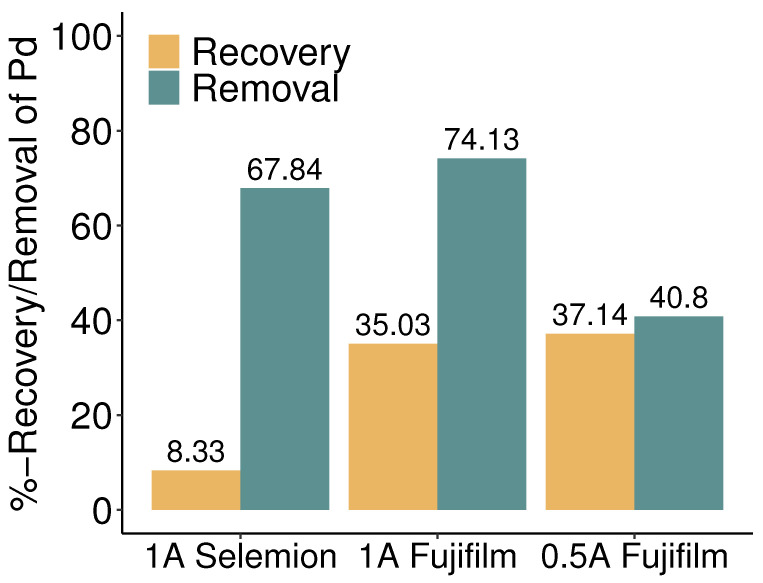
Percentage increase of palladium concentration in the concentrate compartment and decrease of palladium concentration in the diluate compartment. For Fujifilm membranes, the concentration change after two hours of electrodialysis is considered, whereas, for Selemion membranes, the concentration change after the abortion of the experiments after 60 min is considered.

For both sets of membranes, a significant disparity between palladium removal and recovery was observed when working with a current of 1 A, as highlighted in Figure 4. With Selemion membranes, the removal was 60% higher than the recovery, and with Fujifilm membranes, the removal exceeded the recovery by 40%. Subsequently, an additional electrodialysis experiment was carried out using Fujifilm membranes while applying a current of 0.5 A, corresponding to a current density of approximately 14 mA/${\mathrm{cm}}^{2}$. Interestingly, a similar level of palladium recovery was attained with Fujifilm membranes at both 1 A and 0.5 A currents, while at the lower current, the mismatch between removal and recovery was less than 4%.

The reason for performing the additional experiment at 0.5 A only with Fujifilm membranes stemmed from the substantial water drag occurring during electrodialysis with Selemion membranes. Water permeation through the membrane occurs via two mechanisms: osmosis and electroosmosis. Osmosis involves the simple diffusion of water, while electroosmosis entails the transport of water molecules within the hydration shells of counter-ions as they migrate under the influence of the electric field. Studying 20 commercial IEMs, Kingsbury et al. showed that the magnitude of water permeation correlated to the degree of co-ions transport, i.e., salt diffusion, in IEMs, and that these factors were primarily determined by the water and salt diffusion coefficients within the membrane. The commercial IEMs exhibited significant variations in water and salt permeance, spanning several orders of magnitude [40]. In this study, the electroosmotic drag was particularly high when using Selemion membranes. The experiment had to be aborted after 60 min due to the depletion of the dilute solution. In contrast, the Fujifilm membranes are designed for low water permeability [38]. Electrodialysis with these membranes exhibited no significant alteration in volumes between the concentrate and diluate batches. The ion-exchange capacity of Selemion membranes is approximately twice as high as that of Fujifilm membranes, as reported in Table 2. The distinction in ion-exchange capacity indicates a difference in the quantity of ion-exchange sites or functional groups on the membrane’s surface. Low ion-exchange capacity is often associated with reduced hydrophilicity, as there are fewer polar and hydrophilic sites available for interaction with water molecules [45,46]. In a comparative analysis of multiple ion-exchange membranes, Fujifilm Type 12 was characterized as highly crosslinked and displayed the least degree of hydration among the membranes examined [47].

**Figure 5:**
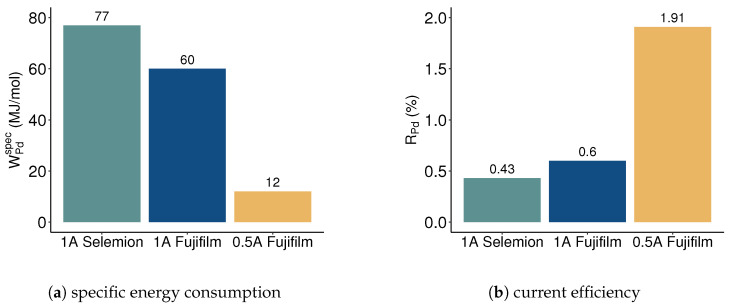
Specific energy consumption per mol of palladium recovered in the concentrate (**a**), and current efficiency for palladium removal (**b**), for Selemion and Fujifilm membranes at different current intensities. For Fujifilm membranes, the work input and concentration change after 2 h of electrodialysis were considered, whereas, for Selemion membranes, the work input and concentration change after the abortion of the experiments (after 60 min) were considered.

**Figure A1:**
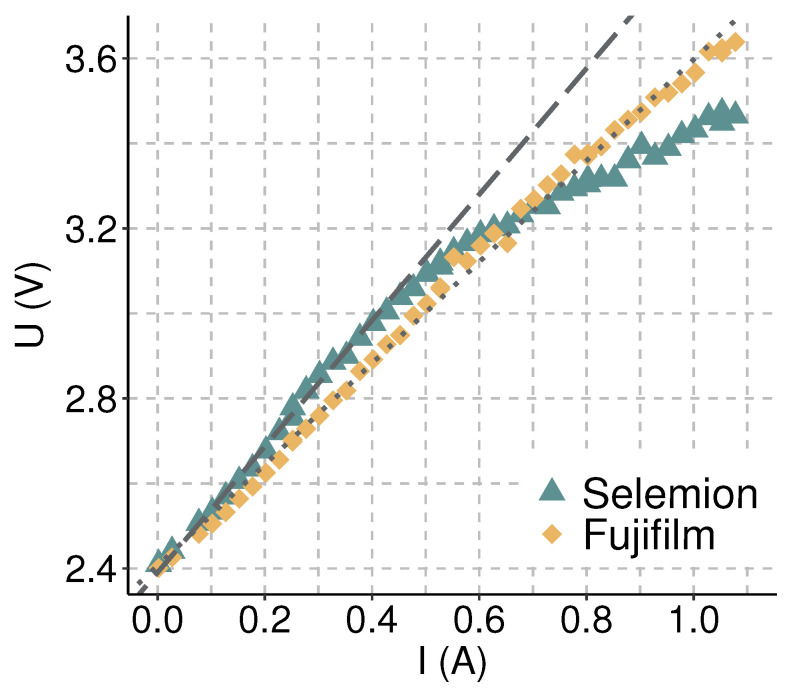
Current-voltage characteristics for Selemion and Fujifilm membranes in the electrodialysis cell. The gray dotted line is a linear approximation for the Fujifilm membranes over the whole range of measurements. The grey dashed line is a linear approximation for the Selemion membranes over the first 14 data points, after which the slope flattened significantly.

Both osmosis and salt diffusion contribute to lowering the salt concentration in the concentrated feed, compromising the effectiveness of the electrodialysis separation processes, as evidenced in this study by the low recovery achieved with Selemion membranes (see Figure 3 and Figure 4). According to the literature, osmosis can reduce the current efficiency in electrodialysis by approximately 10% compared to ideally selective membranes [48], while salt diffusion may elevate the required energy consumption by a factor of 2 to 3 [49]. The specific energy consumption per unit of recovered palladium equivalent and the current efficiency for palladium recovery at the different process conditions applied in this study are shown in Figure 5. The energy consumption per mol of recovered palladium was 60 MJ/mol for Fujifilm membranes at 1 A and scaled down by a factor of five when reducing the current to 0.5 A. With 77 MJ/mol, the palladium-specific energy consumption was highest when utilizing Selemion membranes at 1 A, primarily due to the limited concentration increase. However, operating electrodialysis at overlimiting currents also increases the energy consumption due to the charge transfer occurring in secondary reactions. Throughout the electrodialysis experiments, the measured electrical potentials exhibited stability across all configurations: 3.4 V for Selemion membranes at 1 A, 3.7 V for Fujifilm membranes at 1 A, and 3.1 V for Fujifilm membranes at 0.5 A. Remarkably, the electric potential was similar for Selemion and Fujifilm membranes in the linear current-voltage region (see Figure A1), despite the latter exhibiting a threefold higher membrane resistance. This observation suggests that the membranes did not exert a predominant influence on the stack resistance with the applied operational conditions.

The current efficiency was evaluated as the ratio of the transferred charge that resulted in the recovery of palladium in the concentrate compartment to the overall coulombs transferred in the system. As illustrated in Figure 5b, the current efficiency remained low across all three configurations, peaking at approximately 2% for Fujifilm membranes at 0.5 A. This diminished current efficiency can be attributed to the presence of competing ions responsible for carrying charge within the system, predominantly originating from the hydrochloric acid component.

**Figure 6:**
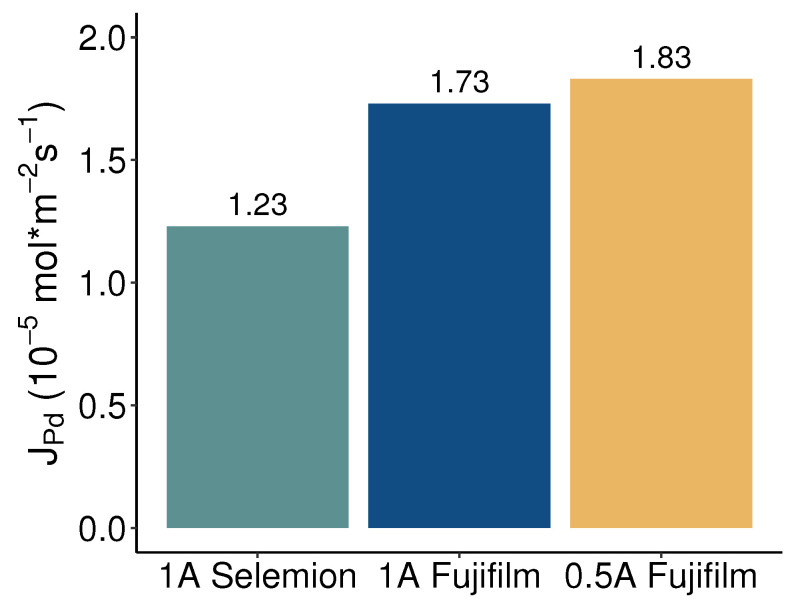
Palladium flux calculated based on the change in Pd concentration in the concentrate compartment for electrodialysis with Selemion and Fujifilm membranes at different current intensities.

**Figure 7:**
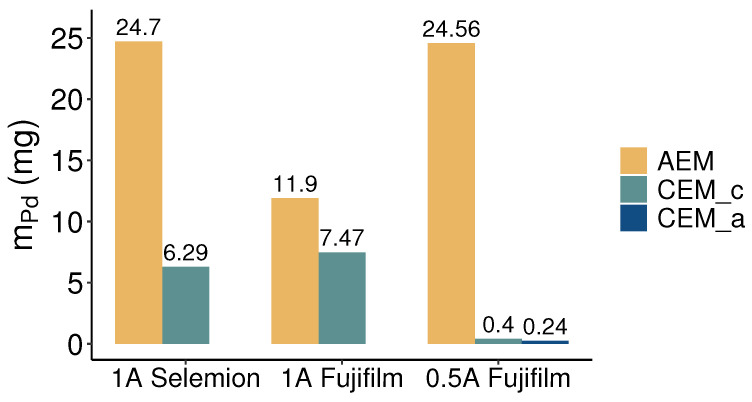
Mass of palladium adhered to the AEMs and CEMs after the electrodialysis experiments. CEM_c and CEM_a are the CEMs at the cathode and anode sides, respectively.

Figure 6 shows the average palladium flux into the concentrate compartment for electrodialysis employing Selemion membranes at a current of 1 A and utilizing Fujifilm membranes at currents of 0.5 A and 1 A. With 1.23$\;\cdot \;10^{-5}$$\mathrm{mol}\;\cdot \;m^{-2}s^{-1}$, the Selemion membranes exhibited the lowest flux due to a modest overall concentration rise of 8% in the concentrate compartment. With Fujifilm membranes, comparable palladium fluxes of 1.73 and 1.83$\;\cdot \;10^{-5}$$\mathrm{mol}\;\cdot \;m^{-2}s^{-1}$ were obtained at currents of 1 A and 0.5 A, respectively. The resembling palladium fluxes at both currents align well with the consistency observed in concentration changes within the concentrate compartments. However, Figure 4 indicates that the number of palladium equivalents leaving the diluate compartment is greater than the palladium flux into the concentrate compartment, especially for the 1 A current. Evidently, a fraction of the palladium extracted from the diluate stream ends up somewhere else than in the concentrate batch. This can be caused by palladium deposition in or on the membranes, palladium precipitation, or leakage of palladium into the rinse solutions, potentially undergoing reduction at the electrodes. All membranes showed brownish-orange decolorization after being subjected to electrodialysis experiments, with a notably higher intensity for the AEMs. The color seemed to originate from within the membrane matrix and could not be washed off with deionized water. No solids were observed in the feed solutions and sample containers during or after the experiments. To clarify the behavior of palladium in the electrodialysis cell, the membranes used in the experiments were analyzed for their palladium content. As shown in Figure 7, both membrane types accumulate palladium in their matrix. When drawing 1 A current, substantial amounts of palladium were detected in both the AEMs and CEMs. Due to the affinity of the palladium tetra- and hexachloric complexes to the cathode, in the first set of experiments performed at 1 A, our analysis solely focused on determining the palladium concentration within the cathode-side CEMs. The palladium mass in the AEMs was higher than that in the CEMs for both membrane types, affirming that the palladium was mainly present in its anionic forms as chloric complexes. However, the CEMs used in the experiments at 1 A also exhibited significant levels of palladium. Nonetheless, the mass of palladium detected in the membranes did not justify the significant mismatch between recovery and removal of palladium. It is therefore probable that palladium leaked into the rinse solution during the electrodialysis treatment with 1 A. The permeation of anions through the CEMs indicates that the applied current was of sufficient magnitude for the cathode’s attraction to outweigh the repulsive impact of the negatively charged CEM. Given the substantial palladium content measured in the CEMs, in the subsequent experiment involving Fujifilm membranes at a current of 0.5 A both CEMs were disintegrated and their palladium content was examined. The amount of palladium detected in the cathode-side CEM was twice that observed in the anode-side CEMs. A significant reduction in the amount of palladium attached to the CEMs was evident when the current was reduced. Following the experiment at 0.5 A, samples from the rinse solutions were analyzed, revealing that the palladium concentration in these rinses fell below the limit of detection. Consequently, the 3.66% deficit in palladium recovery compared to removal, as observed in the 0.5 A experiment with Fujifilm membranes, can be attributed to the 25.2 mg of palladium adhered to the membranes, along with the dilution of the concentrate due to water transport.

The results of the species distribution analyses for palladium, conducted using the *Visual MINTEQ* software, are presented in Appendix B. These analyses indicate that, in the solution composition outlined in Table 2, palladium predominantly exists as the PdCl_4_^2−^ complex, with a lower amount of PdCl_3_^−^ complexes. These findings align well with existing literature sources [11,21,28,30,31,50,51]. However, it is worth noticing that exceeding the limiting current can trigger water splitting, leading to noticeable fluctuations in species concentrations and pH levels, particularly at the surfaces of AEMs [52]. Elevated pH levels at the membrane surfaces have the potential to initiate the formation and precipitation of hydroxide species [23]. Furthermore, uneven distribution of electric potential and localized increases in current densities can induce water splitting at the membrane surfaces [53,54,55,56,57,58]. Factors such as variations in membrane surface topography, membrane clogging, and even scaling phenomena can contribute to these disparities in electric potential [59,60].

When assessing the long-term stability of the membranes in this process, it becomes crucial to investigate the extent to which cations, either confined within or adhering to the membranes, experience either adsorption into the membrane structure or precipitation. While adsorption is a dynamic process that reaches saturation, precipitation can persistently accumulate, leading to increased resistance and diminished permeability [61]. Conducting a thorough examination which involves monitoring pH changes at the membrane surfaces during experiments and analyzing the composition of palladium species retained within the membranes, represents an important direction for future research.

## 4. Conclusions

In this proof-of-concept study, the potential and challenges of using electrodialysis to enhance the concentration of palladium in a residual solution after chemical palladium separation were explored. The industrial solution used in this study contained around 1000 mg/L of palladium dissolved in hydrochloric acid with a pH below 1. The electrodialysis performance was systematically examined using two different sets of ion-exchange membranes and two levels of electric currents. The use of membranes with low permeability for water was beneficial, as it mitigated water transport from the dilute to the concentrate compartment while its effect on the stack resistance was low. Approximately 40% of the palladium was recovered within two hours. Palladium accumulation occurred in all membranes, with anion-exchange membranes showing greater accumulation at lower currents and cation-exchange membranes displaying increased accumulation at higher currents. Regulating the current was crucial to avoid leakage and improve the specific energy consumption for palladium recovery. However, the current efficiency did not exceed 2%, which can be attributed to the abundance of hydrochloric acid in the feed solution. Given the low palladium concentration and the prevalence of other ions, the majority of the electrical charge was carried by these competing ions rather than palladium. pH adjustments before electrodialysis can be a strategy to enhance the current efficiency. Furthermore, to evaluate the upscaling potential of electrodialysis for PGM recovery, it is crucial to perform research on the long-term stability of membranes and thoroughly investigate the impact of membrane properties. Appropriate cleaning procedures and the customization of membrane materials and designs are potential strategies to address challenges related to water transport and scaling. This study highlights the pivotal importance of an informed membrane selection, balancing selectivity, conductivity, and water transport in order to minimize energy losses associated with uncontrolled mixing of the electrolyte solution. The species distribution of palladium adhered to the membranes and pH changes occurring at the membrane surfaces during electrodialysis need to be studied to inform further process optimization.

## Figures and Tables

**Table 1:** Specifications of the working solution used in this study.

Pd (mg/L)	Pt (mg/L)	Rh (mg/L)	Fe (mg/L)	Cl (g/L)	pH
1060	24	<5	13	∼50	0.63

**Table 2:** Chemical, structural, and physical properties of the IEMs used in this work.

Membrane	Thickness Dry, (μm)	Reinforcement	Resistance ${}^{a}$ (Ωcm${}^{2}$)	Permselectivity	Water Permeation ${}^{b}$ (mL/(bar · m${}^{2}\;\cdot \;$ h)	Burst Strength kg/cm${}^{2}$	Ion Exchange Capacity (meq/g)
Fujifilm Type 12, AEM	110	polyolefin	6.0	95 ${}^{d}$	2	373	1.1
Fujifilm Type 12, CEM	110	polyolefin	6.0	99 ${}^{d}$	2.5	373	1.0
Selemion AMVN	100	poly- (vinylchloride) ${}^{c}$	2.0	>95 *^e^*	-	250	2.02 ${}^{c}$
Selemion CMVN	100	poly- (vinylchloride) ${}^{c}$	2.0	>97 *^e^*	-	200	1.89 ${}^{c}$

${}^{a}$ in 0.5 M NaCl. ${}^{b}$ in 0.1–0.7 M NaCl. ${}^{c}$ Data taken from [40]. ${}^{d}$ in 0.05–0.5 M KCl. *^e^* in NaCl. Data are provided by the membrane supplier [38,39] if no other source is indicated.

## Data Availability

The data presented in this study are available on request from the corresponding author. The data are not publicly available to safeguard the interests of K.A. Rasmussen AS, who kindly provided us with the working solution used in this study.

## Appendix A

To assess the current regime where the electrodialysis has been performed, current-voltage characteristics were recorded for both sets of membranes within the electrodialysis cell. A Gamry Interface 5000E potentiostat/galvanostat was utilized in conjunction with the corresponding Gamry software (Gamry Instruments Inc., Warminster, PA, USA). Amperodynamic sweeps were conducted, incrementally increasing the current in steps of 0.025 A. Each specific current value was maintained for 30 s to ensure that a stable voltage was achieved. Measurements of both current and voltage were recorded every 0.25 s. Subsequently, the voltage values were plotted against the respective current values using the experimental data to derive the current-voltage characteristics.

## Appendix B

**Table A1:** Speciation distribution for Pd in the feed solution at different pH in %-concentration.

	pH 0	pH 4	pH 7	pH 14
PdCl_4_^2−^ (%)	99.739	99.794	99.794	99.738
FPdCl_3_^−^ (%)	0.26	0.205	0.205	0.259
